# High-flying precision medicine: Leveraging wearable technology for in-flight emergencies

**DOI:** 10.1371/journal.pdig.0000834

**Published:** 2025-04-24

**Authors:** Daniel Seung Kim, Ewan Webster, Fatima Rodriguez, Eleni Linos

**Affiliations:** 1 Center for Digital Health, Department of Medicine, Stanford University, Stanford, California, United States of America; 2 Division of Cardiovascular Medicine, Department of Medicine, Stanford University, Stanford, California, United States of America; 3 Division of Cardiology, Department of Medicine, University of Washington, Seattle, Washington, United States of America,; 4 Departments of Dermatology and Epidemiology and Population Health, Stanford University, Stanford, California, United States of America; McGill University, CANADA

In-flight medical emergencies (IMEs) in the United States (US) are common; estimated to require consultation with ground-based physicians on 1 in 604 flights between 2008 and 2010 [1]. Flight diversions for an IME are estimated to cost between $15,000 and $893,000 per event [2]. With a rapidly aging population and a continuously growing number of air travel passengers each year, the number of IMEs will increase over time [2]. Despite recent advances in medical technologies, the Federal Aviation Administration (FAA) has not mandated updates for the content of required emergency medical kits aboard US airlines since 2006 [3,4]. In this viewpoint, we argue for the broad use of wearable smart devices to enable improved risk stratification and management of IMEs, and specifically to aid ground-based physicians in determining when to medically divert flights, given the high costs associated with such a decision.

Syncope and presyncope account for the highest proportion of IMEs (30%), followed by respiratory distress (10%), and cardiovascular symptoms (7%–8%) [3,4]. The differential diagnosis for each of these conditions is broad, ranging from benign (vasovagal) to potentially life-threatening (ventricular tachycardia, pulmonary embolism, and/or acute coronary syndrome). While trained medical professionals answering calls for assistance should take targeted histories, their ability to obtain objective data is limited by the FAA-mandated emergency medical kits, which contain only a stethoscope and sphygmomanometer for blood pressure readings. In this context, the recent and rapid development of wearable and smart-device technologies can greatly augment the capabilities of a trained medical professional during an IME (see **Fig 1**).

**Fig 1:**
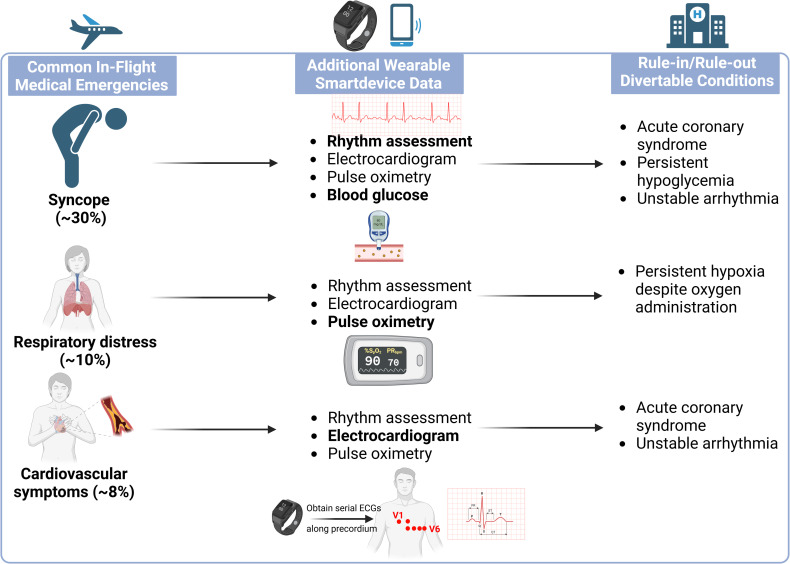
How wearable technology and smart devices can enhance care provided and risk stratification during in-flight emergencies. Created in BioRender. Kim, D. (2025). https://BioRender.com/zrvel1l.

Numerous smart watches can determine electrocardiogram (ECG) rhythm, although only the Apple Watch is approved by the Food and Drug Administration (and only for the diagnosis of atrial fibrillation) [5]. Hence, smartwatches with ECGs can allow for rapid identification of potentially life-threatening cardiac arrhythmias such as atrial fibrillation with rapid ventricular response or ventricular tachycardia. Separately, smartwatches and separate mobile devices such as the AliveCor KardiaMobile are able to record ECGs—which can allow for trained medical professionals without specific training in ECG interpretation to transmit these data to ground-based physicians to aid in risk stratification and medical decision-making on flight diversion. In addition, while not FDA-approved, one study showed that the Apple Watch could be used for the diagnosis of ST-segment changes on ECG [6]. In this study, 54 patients with ST-elevation myocardial infarction had both a standard 12-lead ECG and Apple Watch mediated 9-lead ECG (single lead ECGs across the precordium (V1–V6) and for leads I–III), finding that the Apple Watch had 93% sensitivity and 95% specificity for the diagnosis of ST-elevation myocardial infarction [6]. In addition to ECG capabilities, modern smart watches also frequently come with pulse oximetry capabilities, which can be helpful in the identification and treatment of hypoxia, as well as real-time monitoring of response to limited oxygen supplementation options available (concentrated oxygen is not available in-flight). Finally, with the lack of a glucometer in FAA-mandated emergency medical kits, the potential use of personal continuous glucose monitors (e.g., Dexcom, Freestyle, etc.) can potentially identify hypoglycemia in undifferentiated patients during IMEs and track treatment responses to juice and other remedies available in-flight (summarized in Fig 1).

To date, there is variation in the legal responsibility for off-duty trained medical professionals to respond to IMEs: response is not required in the US, Canada, England, or Singapore, while medical professionals are required to respond in Australia and many European countries. IMEs occurring on flights within or originating from the US are protected by the American Medical Assistance Act (commonly referred to as the “Good Samaritan Act”) [3]. Under the Good Samaritan Act, passengers who provide medical care are protected from liability except in cases of gross negligence or willful misconduct (e.g., rendering care while significantly intoxicated) [4]. However, actively seeking compensation in return for providing aid may jeopardize this immunity [3].

Within this context of legal protection and moral and ethical desire to provide the best care possible for patients with limited resources available, it is our opinion that trained medical professionals should crowd-source wearable technologies (or use their own) during IMEs to gather invaluable objective data that will aid in patient risk stratification and identification of potentially life-threatening conditions that require immediate higher-level of care and flight diversion. While no formal case reports, studies, or meta-analyses exist (to the best of our knowledge), there are numerous anecdotal reports of wearable smart devices being used for the early diagnosis and treatment of hypoxia (via smartwatch pulse oximetry), hypoglycemia (via continuous glucose monitors), arrhythmia (via ECG), and even ST-elevation myocardial infarction (via 6-lead ECG) [7]. Given the high cost of potentially diverting a commercial flight and the inability of ground-based physicians to assess the patient during an IME, it is the ethical and moral responsibility of trained medical professionals responding to an IME to gather as much diagnostic information as possible and advocate for medical flight diversion, when appropriate. With recent technological developments in wearable and personal devices, trained medical professionals now have additional capabilities during IMEs.

As we move toward a fully digital world, trained medical professionals will frequently encounter IMEs where the resources provided are insufficient to provide high-level care. As such, we recommend that all trained medical professionals responding to IMEs leverage smart devices to provide more precision care and risk stratification of patients. Moreover, we note that there remains a lack of formal guidance from the FAA on potential use of wearable smart devices for use in IMEs. Given their ubiquity and multi-faceted capabilities (e.g., ECG, pulse oximetry), we believe that the FAA should release updated guidelines for the use of wearable technologies during IMEs. As well, if the FAA does not update their requirements for emergency medical kits, we would strongly advocate for individual airlines to consider equipping their own planes with mobile technologies that can be used during IMEs. In summary, if we are able to leverage rapid technological developments in the wearable and personal technology space, preferably with formal guidance from the FAA, we believe that precision medicine can be achieved—even at 30,000 feet!

## References

[1] Peterson DC, Martin-Gill C, Guyette FX, Tobias AZ, McCarthy CE, Harrington ST, et al. Outcomes of medical emergencies on commercial airline flights. N Engl J Med. 2013;368(22):2075–83. doi: 10.1056/nejmoa1212052 23718164 PMC3740959

[2] Nascimento IJB do, Jerončić A, Arantes AJR, Brady WJ, Guimaraes NS, Antunes NS, et al. The global incidence of in-flight medical emergencies: a systematic review and meta-analysis of approximately 1.5 billion airline passengers. Am J Emerg Med. 2021 Oct:48:156–64. doi: 10.1016/j.ajem.2021.04.010 33915515

[3] Martin-Gill C, Doyle TJ, Yealy DM. In-flight medical emergencies: a review. JAMA. 2018 Dec 25;320(24):2580–90. doi: 10.1001/jama.2018.19842 30575886

[4] Nable JV, Tupe CL, Gehle BD, Brady WJ. In-flight medical emergencies during commercial travel. N Engl J Med. 2015;373(10):939–45. doi: 10.1056/NEJMra1409213 26332548

[5] Perez MV, Mahaffey KW, Hedlin H, Rumsfeld JS, Garcia A, Ferris T, et al. Large-scale assessment of a smartwatch to identify atrial fibrillation. N Engl J Med. 2019;381(20):1909–17. doi: 10.1056/NEJMoa1901183 31722151 PMC8112605

[6] Spaccarotella CAM, Polimeni A, Migliarino S, Principe E, Curcio A, Mongiardo A, et al. Multichannel electrocardiograms obtained by a smartwatch for the diagnosis of ST-segment changes. 10.1001/jamacardio.2020.3994 PMC7466842 32865545

[7] Lovejoy B. Apple Watch heart attack detection shown to work—but many barriers to real-life use. 15 Aug 2022. [cited 21 Oct 2024] https://9to5mac.com/2022/08/15/apple-watch-heart-attack-detection/

